# Effects of Temporary Functional Deafferentation in Chronic Stroke Patients: Who Profits More?

**DOI:** 10.1155/2018/7392024

**Published:** 2018-07-29

**Authors:** Elisabeth Sens, Marcel Franz, Christoph Preul, Winfried Meissner, Otto W. Witte, Wolfgang H. R. Miltner, Thomas Weiss

**Affiliations:** ^1^Clinical Psychology, Friedrich Schiller University, Jena, Germany; ^2^Department of Neurology, University Hospital, Jena, Germany; ^3^Department of Anesthesiology and Intensive Care, University Hospital, Jena, Germany

## Abstract

Temporary functional deafferentation (TFD) by an anesthetic cream on the stroke-affected forearm was shown to improve sensorimotor abilities of stroke patients. The present study investigated different predictors for sensorimotor improvements during TFD and indicated outcome differences between patients grouped in subcortical lesions only and lesions with any cortical involvement. Thirty-four chronic stroke patients were temporarily deafferented on the more affected forearm by an anesthetic cream. Somatosensory performance was assessed using von Frey Hair and grating orientation task; motor performance was assessed by a shape-sorter-drum task. Seven potential predictors were entered into three linear multiple regression models. Furthermore, effects of TFD on outcome variables for the two groups (cortical versus subcortical lesion) were compared. Sex and sensory deficit were significant predictors for changes in motor function while age accounted for changes in grating orienting task. Males, patients with a stronger sensory deficit, and older patients profited more. None of the potential predictors made significant contributions to changes in threshold for touch. Furthermore, there were no differences in sensorimotor improvement between lesion site groups. The effects of TFD together with the low predictability of the investigated parameters suggest that characteristics of patients alone are not suitable to exclude some patients from TFD.

## 1. Introduction

Impaired somatosensation has been reported to be a predictor for the poor rehabilitation outcome in chronic stroke patients [1]. Over the years, several neurorehabilitative treatment strategies stimulating mechanisms of cortical plasticity have been shown to improve sensorimotor deficits following stroke [2–4]. A new approach demonstrated the benefits on somatosensory sensibility and motor performance of the stroke-affected upper extremity that can arise from a pharmacologically-induced temporary functional deafferentation (TFD) of the forearm [5–7]. TFD might become an additional technique in motor rehabilitation programs such as constraint-induced movement therapy (CIMT) [5–8].

The rationale behind the TFD intervention is based on deafferentation-induced plasticity. It has been shown that deafferentation leads to rapid [9–12] and long-lasting consequences [13–15] in the organization of the primary somatosensory cortex (SI). The neural consequences of deafferentation include disinhibition and hyperactivity of the deafferented neurons due to a lack of excitatory input [16, 17]. This disinhibition renders the deafferented neurons accessible for usually inhibited input from neighboring representations. Exactly, this is the rationale behind the use of TFD in stroke patients. For example, a deafferentation of somatosensory input of relatively less important regions like the middle part of the affected lower arm reduces the excitatory input to its SI representation making it accessible for input from functionally neighboring and, in our example, more important regions like fingers and hand. As has been shown previously [9, 10, 12, 18], the access of fingers and hand to a part of neurons of the representation of the lower arm allows an improvement of the sensitivity of functionally important structures (fingers, hand).

Different variants of TFD have been used up to now including ischemic or pharmacological blockade of peripheral nerve transmission. A commonly used variant of TFD is a tourniquet-induced anesthesia of the right hand which has been shown to improve somatosensory [10, 19] and motor performance [19] of homonym contralateral body parts in healthy subjects as well as in patients with injured nerves [20]. Also in stroke patients, tourniquet-induced anesthesia of the less affected hand results in enhanced motor [21] and sensory [22] abilities. Furthermore, a less painful and unpleasant procedure to induce TFD is using an anesthetic cream. For example, Bjorkman et al. [19] showed an increased capacity in tactile touch perception and two point discrimination in the right hand during TFD by an anesthetic cream on the ipsilateral forearm. Weiss et al. [23] induced TFD by applying an anesthetic cream on the stroke-affected forearm of chronic stroke patients to improve their sensory and motor abilities of the ipsilateral hand. TFD led to a significant improvement of motor performance and somatosensory sensitivity within four hours of TFD on the more affected forearm as compared to the placebo [23]. Additionally, this type of TFD modulates the cortical organization in the contralateral primary somatosensory cortex (SI) [18]. Sens et al. [18] reported a rapid extension of the distance between cortical representations of the stroke-affected thumb and little finger following TFD as well as an increase in the amount of cortical activity of the thumb of the stroke-affected hand during TFD. Moreover, the effects of TFD by an anesthetic cream were different in stroke patients and healthy subjects with improved sensorimotor abilities in the former but not in the latter case [24]. One possible explanation for the different effects of TFD between the groups might be the preexisting deficits in chronic stroke patients due to stroke causing a different efficiency of TFD on the stroke-affected forearm.

The effects of TFD have been attributed to processes of cortical reorganization of the primary motor cortex and SI, for example, reduced interhemispheric inhibition [11, 12, 25], unmasking of existing pathways in SI [18, 26], and invasion of cortical representations by neighboring body parts [12, 18, 27]. TFD with an anesthetic cream is able to modulate mechanisms of cortical reorganization by noninvasive means in stroke patients with different lesion sites [18]. Heretofore, lesion locations and size were less robust predictors of the stroke outcome than clinical factors [28, 29]. Nevertheless, a moderate relationship between lesions of subcortical structures and the poorer short-term outcome of motor recovery after stroke was reported [30]. However, a direct comparison of the effects of TFD between patients with different subcategories of the lesion site has not been drawn, yet.

Strong scientific evidence supports the effects of TFD in improving sensory and motor functions, but the functional characteristics of those who will benefit most from this additional technique are unknown. Therefore, in the present study, we aimed to establish a regression model for patients' somatosensory sensibility and motor capacity during TFD and investigated seven potential predictors (i.e., efficiency of TFD, sensory deficit, side of stroke, chronicity, age, sex, and National Institutes of Health Stroke Scale score). Furthermore, we are also interested in outcome differences between patients with pure subcortical lesions and those with lesions also involving the cortex. A further subdivision of our data was not possible due to the number of patients investigated so far.

## 2. Material and Methods

### 2.1. Patients

We analyzed data of chronic stroke patients reported before in Sens et al. [24] and separated those with only subcortical location of the lesion in one group (*n* = 14) and those with pure cortical or subcortical plus cortical lesions in another group (*n* = 20). We excluded patients with other lesion sites (e.g., paramedian pontine infarction, arteriosclerotic encephalopathy), missing current MRI, and missing values for any predictor. All patients took part in CIMT at our treatment center at the Friedrich Schiller University and volunteered for this study. The procedure of the experiment was described to subjects who were then requested to provide written informed consent prior to their participation. The procedure was conducted in accordance with the Helsinki Declaration on human experimentation and was approved by the ethics committee of the Friedrich Schiller University.

### 2.2. Experimental Design

**Figure 1:**
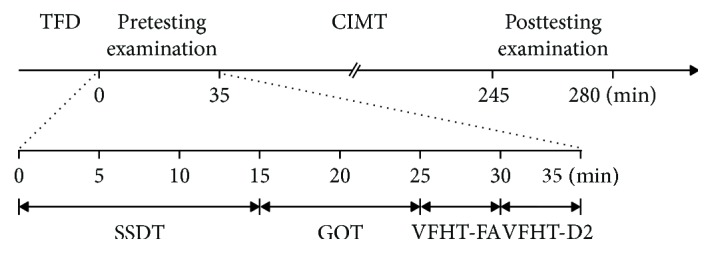
Time line of the experiment and order of examinations. Pre- and posttesting examinations were identical.

During the standard course of CIMT [31] at our treatment center at the Friedrich Schiller University, patients received a pharmacologically-induced TFD by an anesthetic cream on one day. 20 g of EMLA (a eutectic emulsion preparation, containing 2.5% each of lidocaine/prilocaine, AstraZeneca, Sweden) was used per subject to achieve TFD. It took at least 60 minutes to the onset of changes in tactile detection thresholds of the more affected forearm. The cream was applied to the volar side of the more affected forearm covering an area of 50 × 150 mm located about 10 mm above the wrist and sheathed with an occlusive plaster. The anesthetic cream was applied in the morning before the start of the baseline evaluation (t1) of sensory and motor capacity assessment and remained on the arm until the end of the treatment evaluation (t2). Between the two examinations, the patients took part in the usual course of CIMT for 3.5 hours. The same tests outlined below were performed at baseline and treatment evaluations (see Figure 1).

### 2.3. Assessment of Motor and Sensory Functions: Outcome Measures

The methods employed here have been described in detail previously [18, 23].

#### 2.3.1. Shape-Sorter-Drum Task (SSDT)

SSDT was used to measure movement performance with a substantial amount of visual and somatosensory requirements. Subjects were required to take 20 objects from a standard position and to put them into a drum with their more affected hand. Performance time was measured from the start of movements to the instant the last object was put into the drum. The dependent variable, the difference in SSDT performance (ΔSSDT), was defined as the postvalue of SSDT minus the prevalue of SSDT. Since SSDT time is a performance time, lower values indicate a higher improvement of motor performance.

#### 2.3.2. Grating Orienting Task (GOT)

GOT was used to measure limits of spatial resolution of the index finger of the stroke-affected hand, employing a modified technique adapted from Van Boven and Johnson, Bleyenheuft and Thonnard, Tremblay et al., and Craig et al. [32–35]. We used a set of 14 hemispherical plastic domes with gratings cut into their surfaces, resulting in parallel bars and grooves of equal widths (0.5 to 10 mm) on each dome. Gratings were applied with the ridges and grooves randomly oriented in one of two orthogonal directions (perpendicular or parallel to the axis of the finger). Patients were asked to identify the alignment. We determined GOT thresholds, defined as the groove width at which responses were 75% correct. The dependent variable, difference in GOT performance (ΔGOT), was defined as the postvalue of GOT minus the prevalue of GOT. GOT is also a threshold, so lower values indicate a higher improvement of sensory performance.

#### 2.3.3. von Frey Hair Testing (VFHT)

VFHT was used to characterize the limits of tactile detection of the stroke-affected index finger (VFHT-D2). Thresholds for touch were tested at a point marked for VFHT assessment in the middle on the finger pad of the index finger. A von Frey hair set VF2 OptiHair 2 (Marstock Nervtest, Marburg, Germany) was used for assessment. A method of limits was used to determine tactile detection thresholds. A normal distribution of VFHT was achieved by logarithmic transformation of the parameter (log_2_ units) as the forces of von Frey hairs increase by a factor of two [36]. The dependent variable, differences in the performance of VFHT-D2 (ΔVFHT-D2), was defined as the postvalue of VFHT-D2 minus the prevalue of VFHT-D2. Since VFHT characterizes a detection threshold, lower values of ΔVFHT-D2 indicate a higher improvement of sensory performance of the stroke-affected index finger.

The VFHT-D2 was introduced into the investigation slightly after the enrollment of the first patients as an additional assessment of sensory function. Therefore, only 26 patients could be included in the analysis of VFHT-D2.

### 2.4. Potential Predictors

Seven potential predictors were included in the regression models: efficiency of TFD, sensory deficit of the stroke-affected index finger, side of stroke (laterality of hemiparesis), chronicity (time since stroke), age, sex, and the total NIHSS score (stroke severity).

#### 2.4.1. Efficiency of TFD

VFHT was also used to characterize the efficiency of TFD on mechanical thresholds of the more affected forearm. Thresholds for touch were tested at a point marked for VFHT assessment in the center of the occlusive bandage of the treated area of the subjects' stroke-affected forearm. The independent variable, the difference in VFHT at the stroke-affected and deafferented forearm (ΔVFHT-FA), was defined as the log-transformed postvalue of VFHT at the forearm (VFHT-FA) minus the log-transformed prevalue of VFHT-FA. Higher values of ΔVFHT-FA indicate a better efficiency of TFD, that is, less sensitivity. In previous examinations, TFD led to improvements of motor performance and somatosensory sensitivity in the more affected hand of stroke patients [18, 23, 24]. Therefore, the degree of deafferentation might influence the sensorimotor outcomes.

#### 2.4.2. Sensory Deficit of the Stroke-Affected Index Finger

The sensory deficit was defined as the ratio of GOT performance on the index finger for the paretic hand at t1 to GOT performance on the index finger for the control hand. In stroke rehabilitation, sensory impairment represents a well-known predictor of poststroke motor and sensory recovery [1]. A higher sensory loss seems to be associated with a slower sensory [37, 38] and motor [39, 40] improvement. Different degrees of sensory deficits could also be a predictor to characterize those patients who may profit by TFD.

#### 2.4.3. Side of Stroke

The side of stroke is the more affected side of the body caused by stroke. It was obtained by a neurologist after careful consideration of the more affected forearm which was then treated during CIMT. A large quantity of research focuses on differences between functions as well as consequences following stroke of the left or the right hemisphere. Rehabilitation seems to be influenced by laterality of hemiparesis. Patients with a left hemisphere damage used both arms together more often than patients with a right hemisphere lesion, but ipsilesional hand of patients with a left hemisphere is less frequently used [41]. The use of the ipsilesional hand might be influenced by hand preference. Also, sensorimotor improvements and associated cortical changes during TFD could be influenced by the laterality of hemiparesis.

#### 2.4.4. Chronicity

Chronicity was determined as time between the onset of the patients first-ever stroke and their participation in CIMT in years. It is well known that the human brain remains plastic throughout life. Therefore, it is not unexpected that functional improvements are possible at any time after stroke [7, 42]. However, in the course of time, functional as well as structural plasticity occurs in the human brain following stroke and can be colocalized [43]. Thus, effects of TFD could also be influenced by the time since stroke.

#### 2.4.5. Age

The age of patients is often discussed as a predictive factor in stroke rehabilitation; however, reported results are divergent. Numerous studies reported a negative correlation between age and rehabilitation outcome after stroke [38, 44, 45]. Contrary, Wolf et al. [46] found no relationship between age and the effect of CIMT and Rijntjes et al. [42] found a positive correlation. Moreover, touch perception was shown to increase with increasing age of healthy subjects [34] and to be improvable [47]. Older healthy subjects were significantly less sensitive than younger for tactile detection thresholds [48, 49]. Kalisch et al. [50] reported on age-related changes of the hand representation in SI. These findings indicate that age needs to be investigated as a predictor of the sensorimotor outcome during TFD.

#### 2.4.6. Sex

Only a couple of experimental studies have investigated sex differences in stroke rehabilitation. There are a few hints that female patients benefit by stroke rehabilitation treatment less than men [42, 51]. In contrast, Fritz et al. [45] found no relationship between sex and CIMT outcome. Furthermore, tactile spatial acuity differs subtly between the sexes, with women able to indicate finer GOT thresholds detail than men in healthy subjects [52]. Additionally, healthy women were more sensitive than men for tactile detection thresholds [48, 49]. These few hints of gender differences in the stroke rehabilitation outcome and sensory acuity might have an influence of the sensorimotor improvement during TFD.

#### 2.4.7. National Institutes of Health Stroke Scale (NIHSS)

The NIHSS measures impairment and disability of stroke patients and was first designed for use in acute stroke therapy trials [53]. The used version included 15 items and assesses the level of consciousness, extraocular movements, visual fields, facial muscle function, extremity strength, coordination, sensory function, language, speech, and neglect [54]. The NIHSS was obtained at the initial examination of CIMT by a neurologist to describe stroke severity by the total NIHSS score. Stroke severity was identified several times as a predictor of the long-term stroke outcome [55, 56]. Therefore, an initial high total NIHSS score is associated with a worse recovery after stroke.

### 2.5. Statistical Analysis

The potential predictors were entered into three linear multiple regression models using ΔSSDT, ΔGOT, and ΔVFHT-D2 as the dependent variables. A forward stepwise procedure was used in which the least significant variables were removed from the model at each step (entry *p* < 0.05; removal *p* > 0.1). The adjusted *R*^2^ was calculated to assess whether the independent variables were good predictors of the outcome variables. Only significant associations are reported in the final model. Furthermore, to evaluate the effects of TFD on the outcome of ΔSSDT, ΔGOT, and ΔVFHT-D2 within the two groups of the lesion site (subcortical only versus any cortical involvement), the Mann–Whitney *U* test was used because of the different sample sizes. All statistical tests were performed with IBM SPSS Statistic 21.0 for Windows, and the significance level was set to *p* < 0.05.

## 3. Results

### 3.1. Regression Analysis of Predictors

The linear multiple regression models for the three outcome variables and the results for each predictor are presented in Table 1.

#### 3.1.1. Prediction of Changes in Motor Function (ΔSSDT)

**Figure 2:**
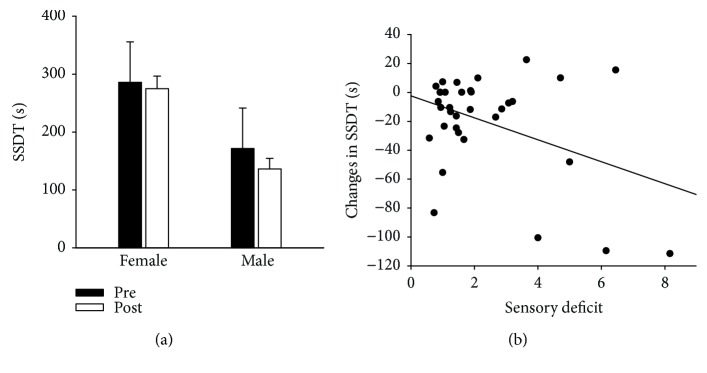
(a) Mean performance time needed and standard errors for the shape-sorter-drum test (SSDT) before (pre) and during (post) temporary functional deafferentation in male (right) and female stroke patients (left). Lower values indicate lower thresholds, that is, better performance. (b) Scatter plot illustrating the relationship (negative correlation) between changes in shape-sorter-drum test (ΔSSDT) scores during temporary functional deafferentation and sensory deficit of the stroke-affected index finger.

The overall model was significant (*F*(2, 31) = 6.696; *p* < 0.004). We found sensory deficit (*B* = −7.853; *p* < 0.01) and sex (*B* = −25.792; *p* < 0.05) as significant predictors for the ΔSSDT model. The adjusted *R*^2^ for the model was 0.257 (nonadjusted *R*^2^ = 0.302); accordingly, 26% of the variance is accounted for the ΔSSDT model. The results show that increased changes in motor performances are associated with male sex (see Figure 2(a)) and stronger sensory deficits (see Figure 2(b)). There was no difference in baseline SSDT between males and females (*z* = −0.41, n.s.). The unique contribution of the sensory deficit (*B* = −7.600; *p* < 0.05) was 14% of the variance of the outcome measurement.

#### 3.1.2. Prediction of Changes in Grating Orienting Task (ΔGOT)

**Figure 3:**
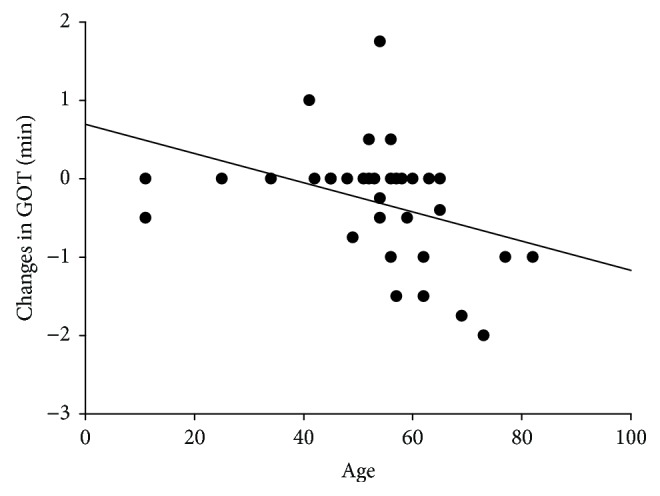
Relationship between age and changes in grating orienting task (ΔGOT) due to temporary functional deafferentation.

The overall model was significant (*F*(1, 32) = 5.491; *p* < 0.025). The only significant predictor for the ΔGOT model was age with a regression coefficient *B* = −0.019 (*p* < 0.05). All other predictors were removed during regression analysis. This model accounted for 12% of the variance in this model (adjusted *R*^2^ = 0.120, nonadjusted *R*^2^ = 0.146). The results indicate that older patients might profit more from TFD with respect to an improvement of somatosensory discrimination (see Figure 3).

#### 3.1.3. Prediction of Changes in Thresholds for Touch (ΔVFHT-D2)

None of the potential predictor variables made significant contribution to the ΔVFHT-D2 model. All predictors were removed during regression analysis. This means that all patients, defined across these seven predictors, benefited equally in changes of tactile detection during TFD.

### 3.2. Outcome Differences between Lesion Site Groups

Lesion site was grouped in subcortical only (14 patients) and any cortical involvement (20 patients). Mean values and standard deviations of raw data for the variables ΔVFHT-FA, ΔVFHT-D2, ΔGOT, and ΔSSDT for both groups are presented in Table 2. We found no significant differences in ΔVFHT-D2 (*z* = −0.39, n.s.), ΔGOT (*z* = −0.29, n.s.), and ΔSSDT (*z* = −0.59, n.s.) between both groups. Patients with only subcortical lesion and patients with any cortical involvement profit both from the effects of TFD.

### 3.3. Supplemental Results

In accordance with the previous publication [24], SSDT, GOT, and VFHT-D2 improved significantly between the baseline and treatment outcomes for the analyzed subgroup. Paired *t*-tests were used to examine performance improvements during TFD for SSDT, GOT, and VFHT-D2 in the partial sample.

We found a significant lower mean performance time (SSDT) during TFD (*M* ± SD: 222.04 ± 262.08) compared with the baseline (242.12 ± 260.49; *t* = 3.38; *p* < 0.05) as well as a significant improvement for the effect of TFD on GOT between treatment evaluation (5.06 ± 3.60) and baseline (5.35 ± 3.41; *t* = 2.25; *p* < 0.05). Both results are in line with our previous investigation [24] including the whole group of stroke patients.

As a precondition for regression analysis of ΔVFHT-D2, a significant improvement between the baseline and treatment outcomes should be demonstrated. In our previous investigation [24] of the whole group of patients, we found a slight but not significant improvement for the effect of TFD on VFHT-D2. Contrary, for the presented patients with only subcortical location of the lesion together with those with pure cortical or subcortical plus cortical lesions, we found a significant lower limit of tactile detection of the stroke-affected index finger during TFD (*M* ± SD: 1.86 ± 4.41) compared with the baseline (1.44 ± 4.15; *t* = 2.15; *p* < 0.05).

## 4. Discussion

In the present study, we used a novel but very simple technique to establish a temporary functional deafferentation (TFD) of the forearm of the more affected arm in poststroke patients by a standard anesthetic cream. This TFD led to a significant increase in motor performance and in somatosensory sensitivity of the affected hand. In the focus of interest, we aimed to investigate predictors of sensorimotor improvements for TFD. We found sex and sensory deficit as significant predictors for changes in motor function. Age was indicated as a significant predictor for changes of grating orienting task. None of the potential predictors made significant contributions to the changes in threshold for touch. Furthermore, there were no differences in sensorimotor improvement between lesion site groups.

TFD by an anesthetic cream led to an improved motor performance as measured by SSDT during TFD in stroke patients. This is in line with previous findings in stroke patients [18, 23, 24]. The most important results were yielded by the predictor analysis of our motor task. In the regression analysis of changes in the shape-sorter-drum task SSDT, the sensory deficit of the stroke-affected index finger and the sex of patients were important predictors of motor improvement during TFD. First, the results show that increased changes in motor performances are associated with male sex (see Figure 2(a)). This is in line with previous reports showing mildly benefits for men in rehabilitation [51, 57]. These authors discussed a sex-related difference in muscle strength, greater in men than in women, as a possible reason for the greater improvement. This difference may even increase in elderly. Additionally, a better speed of performance in different tests of basic motor function was reported in healthy males [58].

Second, increased SSDT motor performance was associated with stronger sensory deficits. Besides pure motor performance, SSDT requires a substantial amount of visual and somatosensory requirements. SSDT was shown to be improved during TFD of the stroke-affected forearm [18, 23]. The influence of sensory function on motor performance and motor learning basing on direct connections between somatosensory and motor cortices is well known [1, 11, 59, 60]. Thus, a reduced sensory input aggravates motor rehabilitation and motor control [39, 40]. TFD in the course of a well-established poststroke rehabilitation program [7, 8] seems to represent a method that might help to improve the motor outcome in patients with sensory deficits even more than patients without sensory deficits. Despite advantages for men and patients with stronger sensory deficit, no prognostic patient characteristics were found for the performance of SSDT. The regression model explains merely a small amount of variation emphasizing that other predictors should be examined.

Moreover, we found no difference between groups with respect to lesion sites. Patients with only subcortical location of the lesions as well as patients with any cortical involvement likewise profit from TFD. Our results are in accordance with previous reports referring no influence of infarct location on recovery after stroke [28, 29, 61].

Somatosensory performance as measured by the grating orienting task (GOT) at the index finger was significantly improved during TFD of the stroke-affected forearm. This result is in accordance with improvements from TFD with an anesthetic cream in stroke patients [18, 23, 24] and healthy subjects [19, 27]. Remarkably, age was indicated as a significant predictor for changes in GOT during TFD. There is an inverse relationship between both variables, that is, as age increases, the predicted limits of spatial resolution of the index finger of the stroke-affected hand (∆GOT) decrease. Thus, older patients might profit more from TFD with respect to an improvement of somatosensory discrimination. This is in contrast to several studies demonstrating a decrease of somatosensory discrimination due to age-related impairments. Thus, the ability to perceive touch applied with von Frey hairs decreases with age as has been shown in several large studies with quantitative sensory testing [48, 49]. Tremblay et al. [34], who applied a very similar method to GOT, showed increased thresholds of somatosensory discrimination with increasing age in healthy subjects. Overall, our results are consistent with previous studies showing a good potential for recovery in older patients [42, 47]. Dinse et al. [47] demonstrated an improvement of tactile acuity in discrimination-impaired elderly induced by a tactile coactivation. Nevertheless, the age of patients explains only 12% of the variance in the regression model for tactile resolution. Taking into account the considerable and significant improvement of GOT by TFD [24], the small amount of variation that can be explained by age alone provides no justification to deny patients TFD.

TFD of the stroke-affected forearm significantly improved the tactile detection thresholds measured with VFHT-D2 for the presented patients with only subcortical location of the lesion together with those with pure cortical or subcortical plus cortical lesions. This is in line with findings in healthy subjects [19]. Contrary, in our previous investigation [24] of the whole group of patients, we did not found a significant improvement for the effect of TFD on the limit of tactile detection of the stroke-affected index finger during TFD. Thus, the effects of TFD with an anesthetic cream on the tactile detection thresholds measured with VFHT-D2 resulted in inconsistent findings. Additionally, none of the potential predictors emerged as a significant predictor for the changes in limits of tactile detection (ΔVFHT-D2). This means that all patients in this separated sample of the two lesion sites, defined across these seven predictors, benefited equally in changes of tactile detection during TFD. Thus, this result suggests that patients with different characteristics might profit from TFD. Nevertheless, it might be necessary to look after other potential predictors which might be more predictive, for example, lesion volume, stroke subtypes, initial impairment, number of stroke, or other general demographics. Moreover, a more detailed differentiation between lesion sites seemed to be necessary. Due to the small group size of other excluded lesion sites (e.g., paramedian pontine infarction), the influence of other lesion sites was not analyzed yet.

## 5. Conclusion

Taken together, our data give a few hints for patients who might profit more than others from a single TFD. Higher age seems to predict higher improvements in spatial resolution by TFD, while stronger sensory deficits and male sex predict higher improvements in the SSDT motor task. However, the fact that the other predictors included had no predictive value for sensorimotor improvements found during TFD implicates that patients' characteristics alone are not suitable to decide who profits more.

## Figures and Tables

**Table 1:** Linear regression models for the outcome variables ∆SSDT, ∆GOT, and ∆VFHT-D2.

Independent variables	∆SSDT Adj. *R*^2^ = 26%		∆GOT Adj. *R*^2^ = 12%		ΔVFHT-D2 —	
	*B* (95% CI)	*p*	*B* (95% CI)	*p*	*B*	*p*
Efficiency of TFD (ΔVFHT-FA)	0.125	0.436	−0.019	0.911	0.028	0.790
Sensory deficit of the stroke-affected index finger	**−7.853 (−13.328, −2.157)**	**0.008**	0.072	0.678	−0.137	0.375
Side of stroke (0 = left; 1 = right)	0.181	0.279	−0.293	0.072	−0.248	0.613
Chronicity	−0.058	0.716	0.084	0.619	−0.005	0.933
Age	0.022	0.890	**−0.019 (−0.035, −0.003)**	**0.025**	−0.014	0.543
Sex (0 = female; 1 = male)	**−25.792 (−46.466, −5.118)**	**0.020**	−0.110	0.515	0.738	0.106
NIHSS	−0.042	0.792	0.182	0.300	0.000	0.636
Constant	8.044	0.394	0.695	0.122	0.435	0.774

ΔSSDT = difference in shape-sorter-drum task (posttest value − pretest value); ΔGOT = difference in grating orientating task (posttest value − pretest value); ΔVFHT-D2 = difference in von Frey hair testing at the index finger (posttest value − pretest value); Adj. *R*^2^ = (adjusted) accounted variance of the model; *B* = regression coefficient; CI = confidence interval for significant regressions; ΔVFHT-D2 = difference in von Frey hair testing at forearm (posttest value − pretest value); NIHSS = National Institutes of Health Stroke Scale.

**Table 2:** Mean and SD of the dependent variables ΔVFHT-FA, ΔSSDT, GOT, and ΔVFHT-D2 for lesion site groups.

	Group	Mean	SD
ΔVFHT-FA	Pure subcortical lesion	2.58	2.26
	Any cortical involvement	4.27	2.00
∆SSDT	Pure subcortical lesion	−21.73	32.70
	Any cortical involvement	−18.93	36.74
∆GOT	Pure subcortical lesion	−0.20	0.91
	Any cortical involvement	−0.36	0.64
∆VFHT-D2	Pure subcortical lesion	−0.38	0.32
	Any cortical involvement	−0.45	1.31

ΔVFHT-FA = difference in von Frey hair testing at forearm (posttest value − pretest value); ΔSSDT = difference in shape-sorter-drum task (posttest value − pretest value); ΔGOT = difference in grating orientating task (posttest value − pretest value); ΔVFHT-D2 = difference in von Frey hair testing at the index finger (posttest value − pretest value).

## Data Availability

The data used to support the findings of this study are available from the corresponding author upon request.

## Abbreviations

CIMT: Constraint-induced movement therapy
D2: Index finger
GOT: Grating orienting task
ΔGOT: Difference in grating orientating task (posttest value − pretest value)
SSDT: Shape-sorter-drum task
ΔSSDT: Difference in shape-sorter-drum task (posttest value − pretest value)
t1: Baseline evaluation
t2: Treatment evaluation
TFD: Temporary functional deafferentation
VFHT: von Frey hair testing
VFHT-D2: von Frey hair testing at the index finger
ΔVFHT-D2: Difference in von Frey hair testing at the index finger (posttest value − pretest value)
VFHT-FA: von Frey hair testing at the forearm
ΔVFHT-FA: Difference in von Frey hair testing at forearm (posttest value − pretest value)
n.s.: Not significant.
